# Exertional Heat Stroke Knowledge and Management among Emergency Medical Service Providers

**DOI:** 10.3390/ijerph18095016

**Published:** 2021-05-10

**Authors:** Rebecca Hirschhorn, Oluwagbemiga DadeMatthews, JoEllen Sefton

**Affiliations:** Warrior Research Center, School of Kinesiology, Auburn University, Auburn, AL 36849, USA; odd0003@auburn.edu (O.D.); jms0018@auburn.edu (J.S.)

**Keywords:** heat-related illness, prehospital, ambulance, paramedic, emergency medical technician

## Abstract

This study evaluated emergency medical services (EMS) providers’ knowledge of exertional heat stroke (EHS) and assessed current EMS capabilities for recognizing and managing EHS. EMS providers currently practicing in the United States were recruited to complete a 25-item questionnaire. There were 216 questionnaire responses (183 complete) representing 28 states. On average, respondents were 42.0 ± 13.0 years old, male (*n* = 163, 75.5%), and white (*n* = 176, 81.5%). Most respondents were Paramedics (*n* = 110, 50.9%) and had ≥16 years of experience (*n* = 109/214, 50.9%) working in EMS. Fifty-five percent (*n* = 99/180) of respondents had previously treated a patient with EHS. The average number of correct answers on the knowledge assessment was 2.6 ± 1.2 out of 7 (~37% correct). Temporal (*n* = 79), tympanic (*n* = 76), and oral (*n* = 68) thermometers were the most prevalent methods of temperature assessment available. Chemical cold packs (*n* = 164) and air conditioning (*n* = 134) were the most prevalent cooling methods available. Respondents demonstrated poor knowledge regarding EHS despite years of experience, and over half stating they had previously treated EHS in the field. Few EMS providers reported having access to an appropriate method of assessing or cooling a patient with EHS. Updated, evidence-based training needs to be provided and stakeholders should ensure their EMS providers have access to appropriate equipment.

## 1. Introduction

Heat stroke is the most severe heat-related illness. Heat stroke is characterized by central nervous system dysfunction (e.g., altered level of consciousness) and elevated core body temperature (>40 °C or 104 °F), and can be categorized as classic or exertional in nature [1,2,3]. Classic heat stroke typically occurs as a passive process where an individual is exposed to a hot environment for a prolonged period of time and is unable to compensate [1]. Conversely, exertional heat stroke (EHS) occurs as a result of excessive metabolic heat production or compromised heat loss during strenuous physical activity that outweighs the individual’s ability thermoregulate [1,3]. Exertional heat stroke and other heat-related illnesses are prevalent among athletes [4,5,6,7], warfighters [8,9,10,11], occupational workers [12] and individuals presenting to emergency departments [13]. There were 34,814 EMS activations for heat-related illnesses in the United States between 2017 and 2018, with 79% documented as heat exhaustion and 17% as heat stroke [14]. The actual proportion of heat stroke cases may be higher, with research reporting approximately 30% of ICD-9 codes of exertional heat illnesses were improperly classified [11].

Timely recognition and treatment is essential for reducing morbidity and mortality of EHS [2,3,15,16,17]. Emergency medical services providers must be able to confidently recognize and manage EHS in the field. Several position and consensus statements on the recognition and management of EHS have been directed towards the sports medicine community [2,3,18]. The 2018 *Consensus Statement- Prehospital Care of Exertional Heat Stroke* [18] communicated best practices for EHS in the prehospital environment and supported evidence-based protocol development to reduce the morbidity and mortality from EHS. These statements agree an accurate core body temperature via rectal thermometry must be obtained as soon as possible and continuously monitored during the treatment process to accurately diagnose EHS [2,3,17,19,20]. Frequently used alternative methods of temperature assessment (oral, temporal, aural thermometry) are too inaccurate to be used to detect heat illness, putting patient lives at risk [19,20,21,22].The gold standard treatment for EHS is cold-water immersion or a similar method achieving a cooling rate of >0.15 °C per minute [2,3,23,24]. Cooling of the patient should occur on-site prior to transport until the patient’s core body temperature reaches 38.9 °C (102 °F). The patient should then be transported to the nearest emergency department via emergency medical services (EMS), eliciting the mantra “cool first, transport second”. Exertional heat stroke is 100% survivable when promptly recognized and proper cooling techniques are implemented [24,25]. A recent survey of EMS medical directors revealed that approximately 15% did not have a protocol specific to the management of EHS and most performed patient cooling en-route to the hospital as opposed to cooling on-site [26].

The primary purpose of the current study was to assess the EHS knowledge among currently practicing EMS providers. A secondary purpose of this study was to assess current EMS capabilities to recognize and manage EHS, as reported by EMS providers.

## 2. Materials and Methods

### 2.1. Study Design and Participants

This study used a cross-sectional design. Participants were asked to complete a short anonymous online questionnaire via Qualtrics (Qualtrics XM, Provo, UT, USA). Currently practicing EMS providers in the United States aged 19 or older where eligible to participate. This study was approved by the Auburn University Institutional Review Board. 

### 2.2. Questionnaire

The 25-item questionnaire was developed specifically for this study and included four sections: (1) demographics; (2) self-rated comfort level with recognizing and managing EHS; (3) EHS knowledge; and (4) experience with EHS and management capabilities (Appendix A). The survey approach used in this study was modeled after a study by Mazerolle, et al. [27] evaluating EHS knowledge among certified athletic trainers. The demographic questions were modeled after a demography study of EMS providers conducted by Rivard, et al. [28] The EHS knowledge section was specifically based on the *Consensus Statement- Prehospital Care of Exertional Heat Stroke* [18]. After the initial questionnaire development, five EMS providers (two paramedics, two advanced EMTs, and one EMT) were asked to pilot the questionnaire and provide feedback. The questionnaire was revised accordingly and finalized by the research team. 

### 2.3. Procedures

A snowball sampling method was used. Contact information for state and regional EMS leadership was gathered using publicly available information found online. An initial email was sent to individuals in state EMS leadership roles asking them to forward the study to their EMS contacts. A follow-up email was sent two weeks after the initial email invitation. Two weeks later an email was sent to individuals in regional EMS leadership roles asking them to forward the study to their EMS contacts. Study flyers were also shared on social media. Questionnaire responses were collected from 12 October 2020 to 4 January 2021. 

### 2.4. Statistical Analysis

Data were exported from Qualtrics into Excel for Microsoft 365 (Version 2102, 2021, Microsoft Corporation, Redmond, WA, USA). Descriptive statistics were calculated for each questionnaire response. A cumulative score was calculated for the EHS knowledge assessment section by totaling the number of correct answers (cumulative knowledge score; CKS), with a maximum possible score of seven. Kruskal-Wallis tests were performed to determine if CKS differed between groups based on state certification level (Paramedic or Other), if additional medical credentials were held (Yes or No), years of experience (<8 years, 8–15 years, ≥16 years), type of position (Career or Volunteer), reported presence of a specific EHS protocol (Yes or No), or self-rated comfort level (Comfortable, Neither, Uncomfortable). An a priori alpha was set at 0.05 for statistical significance. Statistical analysis was performed using SPSS Version 26 (IBM Corp. Released 2019. IBM SPSS Statistics for Windows, Version 26.0. Armonk, NY, USA).

## 3. Results

There were 216 responses to the questionnaire, including incomplete questionnaires. Respondent ages ranged from 19 to 77 (average 42.0 ± 13.0) years old. Most were male (*n* = 163, 75.5%), white (*n* = 176, 81.5%), and had completed a Bachelor’s degree (*n* = 63, 29.2%; Table 1). In total, 28 states are represented, with Alabama (*n* = 41, 19.0%), Hawaii (*n* = 34, 15.7%), and Louisiana (*n* = 48, 22.2%) providing the most responses (Table 2).

Emergency medical technicians (*n* = 77, 35.7%) and paramedics (*n* = 110, 50.9%) were the most prevalent state certification levels (Table 3), with most respondents (*n* = 198, 91.7%) denying additional medical credentials. Approximately 50% of participants reported having at least 16 years of EMS experience (*n* = 109/214, 50.9%). Fire department (*n* = 104/214, 48.6%) and governmental non-fire (*n* = 49/214, 22.9%) were the most common primary agency types reported, with most respondents (*n* = 166/214, 77.6%) working full time for their primary agency. The most common agency types represented were primarily 9-1-1 (*n* = 153/214, 71.5%) and combination 9-1-1 and medical transport (*n* = 46/214, 21.5%).

Approximately half of respondents felt “somewhat comfortable” (*n* = 117/209, 56.0%) and “extremely comfortable” (*n* = 72/209, 34.5%; Table 4) with *recognizing* EHS in the field. The remaining felt “neither comfortable nor uncomfortable” (*n* = 18/209, 8.6%) or “somewhat comfortable” (*n* = 2/209, 1.0%). When asked to rate how comfortable they were with *managing* EHS in the field, most respondents were “somewhat comfortable” (*n* = 110/209, 52.6%) or “extremely comfortable” (*n* = 77/209, 36.8%). The remaining felt “neither comfortable nor uncomfortable” (*n* = 19/209, 9.1%) or “somewhat comfortable” (*n* = 3/209, 1.4%). 

**Figure 1:**
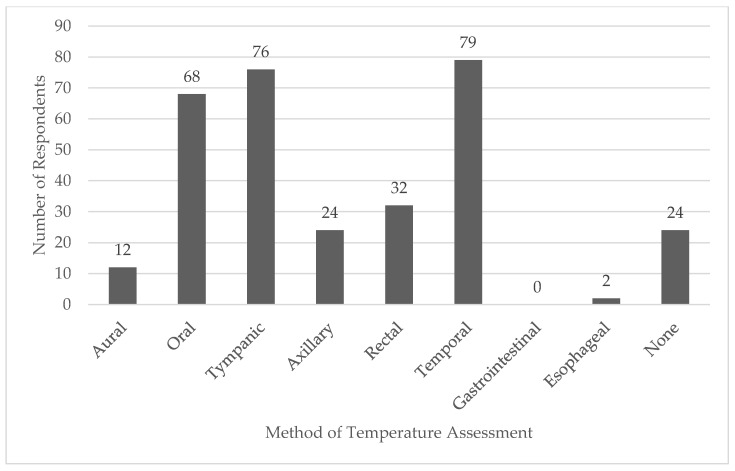
Methods of Temperature Assessment Currently Available to Emergency Medical Services Providers.

**Figure 2:**
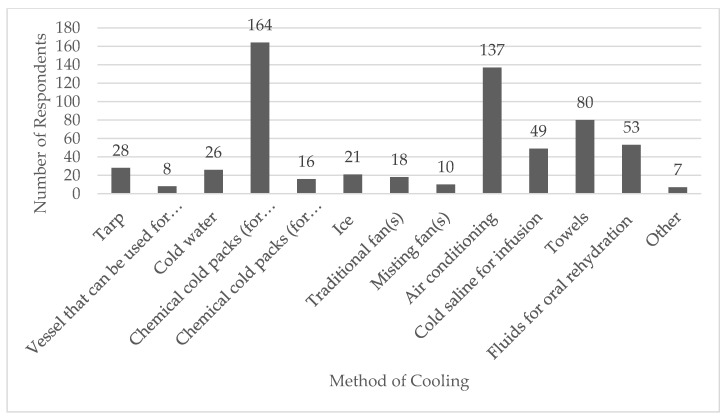
Supplies and Methods for Patient Cooling Currently Available to Emergency Medical Services Providers. Abbreviations: WBI, whole body immersion.

Only 16 out of 216 (8.9%) respondents had previously read the referenced consensus statement. Over half (*n* = 99/180, 55.0%) reported having treated a patient with EHS in the prehospital setting, with 56 (31.1%) reporting they had not treated an EHS patient, and 25 (13.9%) were not sure. Approximately half of respondents (*n* = 92/180, 51.1%) indicated their primary agency had a protocol specifically for EHS while 34.4% (*n* = 62/180) said their agency did not, and 26 (14.4%) were not sure if their agency had an EHS protocol. Respondents indicated temporal (*n* = 79), tympanic (*n* = 76), and oral (*n* = 68) were the most common methods of temperature assessment available on their emergency response vehicle (Figure 1). Chemical cold packs (enough for application of major arteries; *n* = 164) and air conditioning (*n* = 137) were the most commonly available methods of patient cooling on emergency response vehicles (Figure 2).

**Figure 3:**
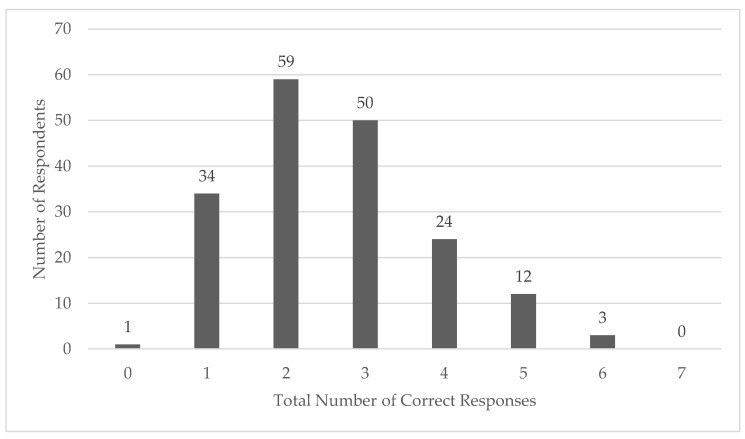
The number of total correct responses to the exertional heat stroke knowledge assessment questions.

There were 183 complete responses for the knowledge assessment questions based on the *Consensus Statement- Prehospital Care of Exertional Heat Stroke* (Table 4) [18]. The average number of correct responses (CKS) was 2.6 ± 1.2 out of 7 questions, or approximately 37% correct (Figure 3). There was a significant difference in CKS between paramedics and other certification levels (H(1) = 6.293, *p* = 0.012). There was not a significant difference in CKS based on having additional medical credentials (H(1) = 0.627, *p* = 0.428), years of experience in EMS (H(2) = 1.074, *p* = 0.584), position type (H(1) = 1.011, *p* = 0.315), or if they reported having a specific EHS protocol at their agency (H(2) = 2.353, *p* = 0.308). There was also no significant difference in CKS based on self-rated comfort level for *recognizing* (H(2) = 1.052, *p* = 0.591) or *managing* (H(2) = 3.542, *p* = 0.170) EHS. 

## 4. Discussion

Most respondents in this study reported they were comfortable recognizing and managing EHS. However, these findings are concerning considering the poor overall knowledge demonstrated in our survey. Few respondents reported having read the *Consensus Statement-Prehospital Care of Exertional Heat Stroke* before this study [18]. Previous research found 25% of EMS medical directors stated that their EMS providers were unaware of the difference between classic heat stroke and EHS [26], demonstrating the need for more education. However, in this same survey, 62% offered continuing education specifically on EHS. Thus, there remains a large discrepancy between continuing education opportunities and medical directors’ confidence in the EMS provider’s knowledge of EHS. It takes years for textbooks and educational content to be updated, thus it is essential that EMS educators and training officers stay up-to-date with current research, especially when position or consensus statements are released. 

Participants in this study demonstrated considerable variability in overall EHS knowledge, with scores ranging from none correct to six out of seven possible correct answers. Responses were scored dichotomously as correct or incorrect; for multi-select style questions, partially correct answers were considered incorrect. There was a statistically significant difference in knowledge between paramedics and other certification levels; however, this may not be a clinically meaningful difference. Both groups generally scored poorly, indicating a need for additional continuing education at all provider levels. Overall, EHS knowledge in recognizing and managing EHS did not differ based on years of experience, additional medical credentials (e.g., ATC, MD, RN), career type, or self-rated comfort levels. However, most of the respondents in our study were career paramedics without additional medical credentials who felt comfortable recognizing and managing EHS. Training officers should consider including regular assessments within their continuing education plans to identify areas where EMS providers may need additional instruction. Future research should investigate the effectiveness of educational interventions for EMS providers on EHS. 

Most EMS providers in this study stated they had treated a patient with EHS in the prehospital setting at some point during their career. A considerable portion were unsure if they had treated a case of EHS, which could be due to poor recall or because they could not differentiate EHS from another heat-related illness such as exertional heat exhaustion. Heat-related illnesses are more frequently encountered by EMS agencies during the summer months of May to September, between 11:00 am and 7:00 pm, and can occur in any geographic region [14,29]. Exertional heat stroke is 100% survivable when recognized and treated promptly and effectively [24,25].

Approximately half of EMS providers in the current study indicated their agency had a protocol for EHS. EMS medical directors largely report having a specific protocol for the management of EHS [26]. However, it is unknown how well these protocols align with current best practices for EHS. The National Association of EMS Physicians encourages incorporating evidence-based prehospital guidelines into EMS systems within 1–2 years from publication and reviewing and revising them every 3–5 years [30].

Emergency medical services providers must be able to distinguish EHS from other heat-related illnesses during patient evaluation. A diagnosis of EHS requires obtaining a core body temperature, preferably via rectal thermometry [2,3,18]. However, only 10% of respondents in this study had access to a rectal thermometer in their emergency response vehicle. Oral, tympanic, and temporal thermometers were the most common devices available for temperature assessment, none of which provide a valid core body temperature in exercising individuals in the heat [20,21,22]. Few EMS medical directors report using rectal thermometry for diagnosing EHS, with oral thermometers used most often [26]. Use of these devices may lead to EMS providers inaccurately ruling out EHS if the temperature is below 40.5 °C. Medical directors should work to align their protocols with best practices and ensure EMS providers working at their agencies have access to a rectal thermometer for the diagnosis of EHS and the training required to accurately use them. Field training officers should include a review of best practices for EHS in their continuing education and ensure all EMS providers are comfortable using a rectal temperature. 

The most common patient cooling methods available in this study were chemical cold packs, air conditioning, and towels. Whole-body cold-water immersion is the ideal cooling method for EHS patients [2,3,18]. There are obvious barriers to achieving whole body immersion en-route to the emergency department since it requires a large tub or tarp and access to ice water. Therefore, “cool first, transport second” is emphasized in EHS management. On-site cooling capabilities should be built into protocols where possible to achieve cooling within 30 min of the collapse [16,18]. Few respondents in this study reported having access to a tarp or other vessel or container for cold-water immersion. Only 21% of EMS medical directors have reported cooling EHS patients on-site, with most cooling patients en-route to the emergency department [26]. If on-site cooling isn’t possible, the most aggressive means of cooling should be used en-route to the emergency department, such as applying cold, wet towels over the patient’s body together with cold-saline infusion and air conditioning [18]. Medical directors should also work with their local emergency departments to set up access to cold-water immersion for EHS patients that arrive and have not been sufficiently cooled. 

This study is the first study to assess EMS provider knowledge of EHS. Similar studies have been conducted surveying athletic trainers who also practice in the prehospital setting [4,27,31,32]. While these studies have demonstrated incomplete adoption of best practices for EHS, the use of cold-water immersion and “cool first, transport second” are common, and adoption has improved over the last several years [4,27,31,32]. Exertional heat stroke is a leading cause of sudden death in sports [33,34]. Considerable emphasis has been placed on implementing best practices for EHS in athletic training since 2002, when the *Inter-association Task Force on Exertional Heat Illnesses Consensus Statement* was published [3,35,36]. Most athletic trainers have also treated at least one case of EHS during their career, with some treating multiple patients within a single football season [27,31]. Lack of administrative support and financial barriers have contributed to the poor adoption of using a rectal thermometer for temperature evaluation in the secondary school setting [37]. Research suggests EMS directors who worked with an athletic trainer were more likely to have implemented best practices for EHS than those who do not [26]. By working together, athletic trainers and EMS agencies may overcome barriers unique to each prehospital setting and improve the continuity of care for EHS patients. Exertional heat illnesses are not limited to the traditional athletic environment where access to an athletic trainer may be available on-site. Therefore, it is vital for EMS agencies to implement an evidence-based EHS protocol to ensure appropriate medical care access. 

A limitation of this study was the small sample size. Due to the snowball sampling method, we could not determine a survey response rate for this study. Most of our respondents were white, male, and had obtained at least some college or a Bachelor’s degree, which is similar to a recent demographic analysis of active EMS providers certified by the National Registry of Emergency Medical Technicians [28]. Additionally, most respondents were Paramedics, had over 15 years of experience working in EMS, worked full time for a fire department who primarily performed 9-1-1 services which were also similar to what has been reported in the literature [28]. Our participants represented 28 states within the United States; however, our results may not represent EMS providers in other states or adequately represent states with low responses overall. We acknowledge that the states with the most responses in our study do not have the most frequent EMS activations for heat-related illnesses; however, our sample is heavily weighted toward the South U.S. Census Region which accounts for approximately 50% of EMS activations for heat-related illnesses [14].

## 5. Conclusions

Emergency medical services providers feel comfortable with recognizing and managing EHS in the field. However, responses demonstrate a poor overall knowledge of EHS best practices in the prehospital setting. Most EMS providers lack access to appropriate equipment for assessing and managing EHS on their emergency response vehicles. Medical directors should continue working with their EMS agencies to develop and implement evidence-based protocols specific to EHS. Continuing education on EHS is needed at all EMS provider levels and should include reviewing the most recent consensus statement. 

## Figures and Tables

**Table 1:** Demographic Information for Questionnaire Participants (*n* = 216).

Variable	*n* (%)
**Age (mean, SD, [range])**	42.0 (13.0), [19–77]
**Gender**	
Male	163 (75.5)
Female	53 (24.5)
Non-binary	0 (0.0)
Prefer Not to Identify	0 (0.0)
**Race/Ethnicity**	
White	176 (81.5)
Asian	12 (5.6)
Black or African American	1 (0.5)
Hispanic or Latino	4 (1.9)
American Indian or Alaskan Native	1 (0.5)
Native Hawaiian or Other Pacific Islander	7 (3.2)
More Than One Race/Ethnicity	10 (4.6)
Prefer Not to Identify	5 (2.3)
**Highest Level of Education Completed**	
High School/GED	6 (2.8)
Some College	60 (27.8)
Associate Degree	57 (26.4)
Bachelor’s Degree	63 (29.2)
Master’s Degree	25 (11.6)
Doctorate Degree	5 (2.3)

Abbreviations: SD, standard deviation; GED, general education diploma.

**Table 2:** States Represented by Participants (*n* = 216).

State	*n* (%)	State	*n* (%)
Alabama	41 (19.0)	Missouri	1 (0.5)
Alaska	3 (1.4)	Montana	3 (1.4)
Arizona	9 (4.2)	New Hampshire	3 (1.4)
Arkansas	7 (3.2)	New Jersey	4 (1.9)
Colorado	12 (5.6)	New Mexico	1 (0.5)
Connecticut	1 (0.5)	New York	1 (0.5)
Florida	1 (0.5)	Pennsylvania	1 (0.5)
Hawaii	34 (15.7)	Rhode Island	1 (0.5)
Idaho	1 (0.5)	South Carolina	9 (4.2)
Illinois	6 (2.8)	South Dakota	10 (4.6)
Iowa	1 (0.5)	Texas	1 (0.5)
Kentucky	4 (1.8)	Vermont	1 (0.5)
Louisiana	48 (22.2)	Virginia	1 (0.5)
Maryland	10 (4.6)	Wisconsin	1 (0.5)

**Table 3:** Participant Emergency Medical Services Experience and Employment Settings.

Variable	*n* (%)
**State Certification Level (*n* = 216)**	
EMR	3 (1.4)
EMT	77 (35.7)
AEMT	20 (9.3)
Paramedic	110 (50.9)
Other	6 (2.8)
**Years of EMS Experience (*n* = 214)**	
2 Years or Less	18 (8.3)
3–7 Years	35 (16.4)
8–15 Years	52 (24.3)
16 Years or More	109 (50.9)
**Type of Position (*n* = 214)**	
Volunteer-Compensated	5 (2.3)
Volunteer- Non-compensated	22 (10.3)
Career-Part-time	21 (9.8)
Career-Full-time	166 (77.6)
**Type of Agency (*n* = 214)**	
Fire Department	104 (48.6)
Private	30 (14.0)
Governmental Non-fire	49 (22.9)
Hospital	11 (5.1)
Volunteer/Rescue Squad	7 (3.3)
Other	13 (6.1)
**Type of Agency Service (*n* = 214)**	
Primarily 9-1-1	153 (71.5)
Combination 9-1-1 and Medical Transport	46 (21.5)
Primarily Medical Transport (Convalescent)	3 (1.4)
Clinical Services	3 (1.4)
Other	9 (4.2)

Abbreviations: AEMT, advanced emergency medical technician; EMR, emergency medical responder; EMS, emergency medical services; EMT, emergency medical technician.

**Table 4:** Exertional Heat Stroke Knowledge Assessment (*n* = 183).

Question and Response Options	*n* (%)	Correct Responses (*n*, %)	Incorrect Responses (*n*, %)
**True or False. Exertional heat stroke is considered a life-threatening medical emergency.**			
True *	181 (98.9)	181 (98.9)	2 (1.1)
False	2 (1.1)		
**Please select the two primary signs and symptoms that characterize exertional heat stroke.**			
Hot, dry skin	103 (27.3)	50 (27.3)	133 (72.7)
Cool, clammy skin	11 (2.9)		
Unconsciousness	19 (5.0)		
Elevated core body temperature (>40.5 °C or 105 °F) *	102 (27.1)		
Profuse sweating	5 (1.3)		
CNS dysfunction (e.g., confusion, altered mental status) *	120 (31.8)		
Vomiting	10 (2.7)		
Dehydration	7 (1.9)		
**What is the most acceptable method of temperature assessment for the diagnosis of exertional heat stroke in the prehospital setting?**			
Aural	4 (2.2)	62 (33.9)	121 (66.1)
Oral	43 (23.5)		
Tympanic	31 (16.9)		
Axillary	19 (10.4)		
Rectal *	62 (33.9)		
Temporal	22 (12.0)		
Gastrointestinal	1 (0.6)		
Esophageal	1 (0.6)		
**Rapid cooling of a patient with exertional heat stroke should occur within ___ minutes from the time of collapse.**			
15 min	152 (83.1)	30 (16.4)	153 (83.6)
30 min*	30 (16.4)		
45 min	0 (0.0)		
60 min	1 (0.6)		
**True or False. A patient with exertional heat stroke should be transported by EMS to the hospital immediately upon EMS arrival and assessment on scene, regardless of any treatments initiated and being provided on-scene.**			
True	118 (64.5)	65 (35.5)	118 (64.5)
False *	65 (35.5)		
**Select all appropriate methods of cooling for the management of exertional heat stroke.**			
Tarp-assisted cooling *	33 (4.1)	0 (0.0)	183 (100.0)
Cold-water immersion (from neck down) *	62 (7.8)		
Ice packs on major arteries	153 (19.1)		
Ice packs on whole body	28 (3.5)		
Ice-water immersion *	38 (4.8)		
Fanning the patient	81 (10.1)		
Move the patient to an area with air conditioning	142 (17.8)		
Cold-water dousing with ice massage	21 (2.6)		
Intravenous cold saline infusion	73 (9.1)		
Placing wet towels on the patient	111 (13.9)		
Providing the patient with oral fluids for rehydration	58 (7.3)		

Abbreviations: CNS, central nervous system; EMS, emergency medical services. Note: Correct answers are denoted with a *. Individual responses to each question were coded as correct or incorrect.

## Data Availability

The data presented in this study are available on request from the corresponding author.

## Appendix A

**Emergency Medical Services Knowledge of Exertional Heat Stroke Questionnaire**

Start of Block: Demographic Information

This first section asks demographic questions about you and your EMS experience.

*What is your age?*

________________________________________________________________

*What is your gender?*

Male

Female

Non-binary

Prefer not to identify

*What is your race/ethnicity?*

White

Asian

Black or African American

Hispanic or Latino

American Indian or Alaskan Native

Native Hawaiian or Other Pacific Islander

More than one race/ethnicity

Prefer not to identify

*What is the highest level of education you have completed?*

High School/GED

Some college

Associate’s degree

Bachelor’s degree

Master’s degree

Doctorate degree

*In what state (including the District of Columbia) do you currently practice in?*

Alabama

Alaska

Arizona

Arkansas

California

Colorado

Connecticut

Delaware

District of Columbia

Florida

Georgia

Hawaii

Idaho

Illinois

Indiana

Iowa

Kansas

Kentucky

Louisiana

Maine

Maryland

Massachusetts

Michigan

Minnesota

Mississippi

Missouri

Montana

Nebraska

Nevada

New Hampshire

New Jersey

New Mexico

New York

North Carolina

North Dakota

Ohio

Oklahoma

Oregon

Pennsylvania

Rhode Island

South Carolina

South Dakota

Tennessee

Texas

Utah

Vermont

Virginia

Washington

West Virginia

Wisconsin

Wyoming

*What is your state certification level?*

Emergency Medical Responder

Emergency Medical Technician

Advanced Emergency Medical Technician

Paramedic

Other

*7a. Do you have any other medical credentials (e.g., MD, DO, RN, ATC)?*

Yes

No

*7b. Please list your current medical credentials in addition to your EMS certification.*

________________________________________________________________

*How many years of experience do you have working in EMS?*

2 years or less

3–7 years

8–15 years

16 or more

*What type of position do you have at your agency (your primary agency, if working at more than one)?*

Volunteer-Compensated

Volunteer-Non-compensated

Career-Part-time

Career-Full-time

*What type of agency do you work for (your primary agency, if working at more than one)?*

Fire department

Private

Governmental Non-Fire

Hospital

Volunteer/Rescue Squad

Other

*What type of services does your agency provide (your primary agency, if working at more than one)?*

Primarily 9-1-1

Combination of 9-1-1 and medical transport

Primarily medical transport (convalescent)

Clinical services

Other

End of Block: Demographic Information

Start of Block: Self-Rated Comfort Level

This section asks you about how comfortable you are recognizing and managing exertional heat stroke in the field.

*Please rank how comfortable you are with recognizing exertional heat stroke in the field.*

Extremely comfortable

Somewhat comfortable

Neither comfortable nor uncomfortable

Somewhat uncomfortable

Extremely uncomfortable

*Please rank how comfortable you are with managing exertional heat stroke in the field.*

Extremely comfortable

Somewhat comfortable

Neither comfortable nor uncomfortable

Somewhat uncomfortable

Extremely uncomfortable

End of Block: Self-Rated Comfort Level

Start of Block: EHS Knowledge

This section asks you questions specifically about exertional heat stroke. Please answer each question to the best of your ability.

*True or False. Exertional heat stroke is considered a life-threatening medical emergency.*

True

False

*Please select the two primary signs and symptoms that characterize exertional heat stroke.*

Hot, dry skin

Cool, clammy skin

Unconsciousness

Elevated core body temperature (>40.5 C or 105 F)

Profuse sweating

Central nervous system dysfunction (e.g., confusion, altered mental status)

Vomiting

Dehydration

*What is the most acceptable method of temperature assessment for the diagnosis of exertional heat stroke in the prehospital setting?*

Aural

Oral

Tympanic

Axillary

Rectal

Temporal

Gastrointestinal

Esophageal

*Rapid cooling of a patient with exertional heat stroke should occur within __ minutes from the time of collapse.*

15 min

30 min

45 min

60 min

*True or False. A patient with exertional heat stroke should be transported by EMS to the hospital immediately upon EMS arrival and assessment on scene, regardless of any treatments initiated and being provided on-scene.*

True

False

*Select all appropriate methods of cooling for the management of exertional heat stroke.*

Tarp-assisted cooling

Cold-water immersion (from neck down)

Ice packs on major arteries

Ice packs on whole body

Ice-water immersion

Fanning the patient

Move the patient to an area with air conditioning

Cold-water dousing with ice massage

Intravenous cold saline infusion

Placing wet towels on the patient

Providing the patient with oral fluids for rehydration

*Cooling of a patient with exertional heat stroke should continue until ___.*

The patient begins to shiver

The patient regains consciousness

The patient’s core body temperature reaches 38.6 °C or 102 °F

The patient has been cooled for 10 min

EMS have arrived on-scene

EMS have arrived at the emergency department with the patient

End of Block: EHS Knowledge

Start of Block: Experience with EHS

This section asks questions about your experience with exertional heat stroke and the treatment capabilities you have available on your EMS unit at your primary agency.

*Have you ever read the document “Consensus Statement-Prehospital Care of Exertional Heat Stroke” published in Prehospital Emergency Care?*

Yes

No

*Have you ever treated a patient with exertional heat stroke in the prehospital setting?*

Yes

No

I’m not sure

*Does your primary EMS agency have a protocol specifically for exertional heat stroke?*

Yes

No

I’m not sure

*What methods of temperature assessment do you have available in your emergency response vehicle? (select all that apply)*

Aural

Oral

Tympanic

Axillary

Rectal

Temporal

Gastrointestinal

Esophageal

None

*What supplies/methods of patient cooling do you have available to you in your emergency response vehicle? (select all that apply)*

Tarp

Vessel or container that can be used for whole-body immersion (e.g., body bag, commercially available device)

Cold water

Chemical cold packs (enough for application on major arteries)

Chemical cold packs (enough for application to whole body)

Ice (for ice bags or ice water)

Traditional fan(s)

Misting fan(s)

Air conditioning

Cold saline for infusion

Towels

Fluids for oral rehydration (i.e., water, sports drink)

Other

End of Block: Experience with EHS
